# Effect of morphine-induced postconditioning in corrections of tetralogy of fallot

**DOI:** 10.1186/1749-8090-8-76

**Published:** 2013-04-11

**Authors:** Rufang Zhang, Li Shen, Yewei Xie, Lin Gen, Xiaobing Li, Qiang Ji

**Affiliations:** 1 Department of Cardiothoracic Surgery, Shanghai Children’s Hospital, Shanghai Jiaotong University, Shanghai, 1400 Western Beijing Rd, Shanghai 200040, People's Republic of China; 2 Department of Thoracic Cardiovascular Surgery of Tongji Hospital of Tongji University, Shanghai, 389 Xincun Rd, Shanghai 200065, People's Republic of China

**Keywords:** Pharmacological postconditioning, Morphine, Ischemia reperfusion injury, Pediatric cardiac surgery, Trials

## Abstract

**Background:**

Results of previous reports on ischemic postconditioning in animals and humans were very encouraging. Although ischemic postconditioning possessed a wide prospect of clinical application, debates on the precise ischemic postconditioning algorithm to use in clinical settings were ongoing. In this regard, pharmacological strategies were possible alternative methods. Accumulating data demonstrated that pharmacological postconditioning with morphine conferred cardioprotection in animals. This trial aimed to evaluate the effect of morphine-induced postconditioning on protection against myocardial ischemia/reperfusion injury in patients undergoing corrections of Tetralogy of Fallot (TOF).

**Methods:**

Eight-nine consecutive children scheduled for corrections of TOF were enrolled and randomly assigned to either a postconditioning group (patients received a dose of morphine (0.1 mg/kg) injected via a cardioplegia needle into the aortic root for direct and focused delivery to the heart within 1 minute starting at 3 min before aorta cross-clamp removal, n=44) or a control group (the same protocol was performed as in the postconditioning group except that patients received the same volume of saline instead, n=45). The peri-operative relevant data were investigated and analyzed, and the cardiac troponin I (cTnI) was assayed preoperatively, and then 4 h, 8 h, 12 h, 24 h and 48 h after reperfusion.

**Results:**

Morphine-induced postconditioning reduced postoperative peak cTnI release as compared to the control group (0.57 ± 0.15 versus 0.75 ± 0.20 ng/mL, p<0.0001). Morphine-induced postconditioned patients had lower peak inotropic score (5.7 ± 2.4 versus 8.4 ± 3.6, p<0.0001) and shorter duration of mechanical ventilation as well as ICU stay (20.6 ± 6.8 versus 28.5 ± 8.3 hours, p<0.0001 and 40.4 ± 10.3 versus 57.8 ± 15.2 hours, p<0.0001, respectively), while higher left ventricular ejection fraction as well as cardiac output (0.57±0.15 versus 0.51±0.13, p=0.0467 and 1.39 ± 0.25 versus 1.24 ± 0.21 L/min, p=0.0029, respectively) as compared to the control group during the first postoperative 24 hours.

**Conclusions:**

Morphine-induced postconditioning may provide enhanced cardioprotection against ischemia/reperfusion injury in children undergoing corrections of TOF.

## Background

Although the application of cardioplegia is an effective strategy for cardio-protection, it does not completely eradicate myocardial ischemia/reperfusion injury due to cardiac arrest during cardiac surgery. It was well-established that ischemic preconditioning, which involved a series of brief ischemia/reperfusion cycles before index ischemia, was explicitly cardioprotective strategy [1-5]. Unlike preconditioning, ischemic postconditioning, which involved brief episodes of ischemia/reperfusion during early reperfusion, did not require initiation before the ischemic event [1-8]. And hence, ischemic postconditioning was easily performed in all cardiac operations such as in those for severe aortic valve insufficiency or aortic valve stenosis where preconditioning often was difficult to be carried out before operation due to possible heart arrest and distension induced during repeated aortic clamping [1-8]. Results of previous reports on ischemic postconditioning were very encouraging in animals and humans [1-10].

Although ischemic postconditioning possessed a wide prospect of clinical application, debates on the precise ischemic postconditioning algorithm to use in clinical settings were ongoing. In this regard, pharmacological strategies [11,12], which would avoid some adverse consequences associated with intermittent cross-clamping (such as aortic injury, left ventricular distention, and malignant arrhythmia), were possible alternative methods. Morphine, a classic opioid drug, was particularly interesting cardioprotective ligands to anesthesiologists and clinicians due to their widely used perioperatively. Schultz’s group first reported that morphine-induced preconditioning mimicked ischemic preconditioning [13]. Accumulating data demonstrated that morphine-induced postconditioning provided powerful cardioprotective effect, which seemed to be similar to ischemic postconditioning [12,14-17]. So far, the vast majority of previous studies assessed the impact of morphine-induced postconditioning on cardioprotection in animal experiments. However,there were few reports focused on morphine-induced postconditioning in the field of cardiac surgery, especially pediatric cardiac surgery [12,18]. Although major advances have been made in the field of cardiac surgery for Tetralogy of Fallot (TOF), inadequate myocardial protection may remain a persistent source of mortality and morbidity associated with surgery for TOF [19]. Whether morphine-induced postconditioning has an additive effect on cardioplegia or not in the setting of pediatric cardiac surgery for TOF remains to be determined.

The purpose of this study was to test the hypothesis that morphine-induced postconditioning could provide enhanced cardio-protection against ischemia/ reperfusion injury in children undergoing corrections of TOF.

## Methods

### Subjects

Eligible patients were less than 18 years of age, scheduled for total correction of TOF, and cardiac function NYHA class I-III.

Exclusion criteria were as follows: TOF with pulmonary atresia, TOF with absent pulmonary artery, concomitant infective endocarditis, prior palliation with a systemic-to-pulmonary artery shunt, postoperative residual ventricular septal defect, and postoperative moderate and severe pulmonary valve regurgitation.

All selected patients underwent preoperative electrocardiogram, chest X-ray film and echocardiography. None of selected patients received preoperative inotropic support. The guardians of selected patients signed an informed consent approved by the ethics committee.

### Grouping

This study protocol was approved by the ethics committee of Tongji hospital affiliated to Tongji University and was consistent with the *Declaration of Helsinki*.

All selected patients were divided randomly into either a postconditioning group or a control group through a computer-generated randomization sequence. The randomization assignment for each patient was kept in a sealed envelope which was opened by a trial investigator at the time of patient enrollment. The hospital staff caring for postoperative patients was blinded for group allocation.

In the postconditioning group, starting at 3 min before aorta cross-clamp removal, patients received a dose of morphine (0.1 mg/kg) injected via a cardioplegia needle into the aortic root for direct and focused delivery to the heart within 1 minute. In the control group, the same protocol was performed as in the postconditioning group except that patients received the same volume of saline instead.

With reference to previous reports [20,21], in which 0.1 mg/kg was indicated as better dosage for morphine postconditioning, we used the dosage for morphine postconditioning in this trial.

### Clinical data

The pre-, intra- and post-operative relevant data of all selected patients were investigated and analyzed. Preoperative information included age, gender, weight, McGoon ratio, left ventricular end-diastolic volume index (LVEDVI), left ventricular ejection fraction (LVEF), cardiac output, hemoglobin, and saturation of arterial blood oxygen (in absence of supplemental oxygen administration). Intraoperative variables included operative type (transannular patches or not), duration of cardiopulmonary bypass (CPB) and aortic cross clamping (ACC), and resumption of cardiac activity/rhythm. Postoperative data included the first postoperative 24-hour parameters (such as urine volume, drainage volume, blood transfusion volume, peak inotropic score, creatinine, use of furosemide as well as its intravenous infusion dose), duration of mechanical ventilation and intensive care unit (ICU) stay, and in-hospital mortality. Echocardiography was performed to assess LVEF and cardiac output during the first postoperative day and before discharge. All patients were followed up for at least 30 days following surgery.

The McGoon ratio is calculated by dividing the sum of the diameter of the bilateral central pulmonary arteries by the diameter of the descending aorta at the level of the diaphragm. The first postoperative 24-hour quantitative measures of inotropes were evaluated by the inotropic score according to the following formula [22]: ([dopamine + dobutamine] ×1) + (milrinone × 15) + ([epinephrine + norepinephrine + isoprenaline] ×100).

Management of inotropic dose was according to hemodynamic stability as reflected by such parameters as arterial blood pressure, central venous pressure, etc. Dopamine was employed as the first-line inotrope with epinephrine and/or norepinephrine as second-line inotrope for hemodynamic support in cases of hemodynamic instability as reflected by systemic blood pressure inadequacy despite maximal fluid management and dopamine use. Weaning from mechanical ventilation depended on hemodynamics, arterial blood gas analysis, etc. to assess for cardiopulmonary function; no excessive bleeding and a mandatory chest radiograph before extubation to rule out pneumothorax, pleural effusion and atelectasis. Criteria for leaving ICU included an alert, hemodynamically stable patient with an inotropic score less than 5, better pulmonary function with no metabolic acidosis, urine output more than 0.5 ml/kg/h and a mandatory chest radiograph before leaving ICU to rule out pneumothorax, pleural effusion and atelectasis. The indications that guided the use of furosemide included oliguria (urine output <0.5 ml/kg/h) and development of hypervolemia [8].

### Blood sampling and biochemical analysis

For each patient, 2 ml blood samples were collected preoperatively, and then 4 hours, 8 hours, 12 hours, 24 hours, and 48 hours after aorta cross-clamp removal for determination of the plasma levels of cardiac troponin I (cTnI). The blood samples were transferred into dry glass tubes and stored at 4°C until centrifugation. Plasma separated after centrifugation was frozen at −80 C until assayed for cTnI using the cTnI ELISA kit which was obtained from Jidan Biotechnology Company (Nanjing, China). The cTnI values were expressed as ng/ml (normal reference value in our hospital: <0.15 ng/mL). The cTnI concentration was measured in the clinical laboratory of our hospital by individuals unaware of the group allocation.

Total cTnI release in the control and postconditioning group was represented as an area under curve (AUC) from preoperation (C_0_) to the last sampling time (C_48_). It was calculated using the equation for determining trapezoid area as follows [8]: AUC_0-48_ = [(C_0_+C_4_) ×4+ (C_4_+C_8_) ×4+ (C_8_+C_12_) ×4+ (C_12_+C_24_) ×12+ (C_24_+C_48_) ×24] /2. Total cTnI release from C_0_ to C_8_ and C_8_ to C_48_ represented as AUC_0-8_ and AUC_8-48_ respectively, were calculated accordingly.

### Operation

During the surgical procedure, anesthesia was induced with midazolam (0.05 mg/kg), fentanyl (4 μg/kg) and vecuronium (0.1 μg/kg), and maintained with intravenous fentanyl (total amount of fentanyl was less than 15 μg/kg during operation). All the operations were performed by the same surgical team. The operations were performed using moderate hypothermic cardiopulmonary bypass (28-31°C) with bicaval cannulation and left heart venting via the right superior pulmonary vein. The myocardium was protected using intermittent perfusion of cold blood cardioplegic solution at 4°C and in a ratio of 1:4. The cold blood cardioplegia which was composed of sodium chloride at 132 mmol/L, potassium chloride at 16 mmol/L, calcium chloride at 1.8 mmol/L, magnesium sulphate at 15 mmol/L, procaine at 0.05 mmol/L, and sodium bicarbonate at 19 mmol/L, was infused into the aortic root if there was no aortic valve regurgitation. For patients with aortic regurgitation, the aorta was opened promptly after the cross-clamp was applied, the left and right coronary artery ostia were cannulated, and the cold blood cardioplegia was delivered though a Y bifurcated catheter. The initial dose of cardioplegia infused was at 20 ml/kg; thereafter, the cardioplegia solution was re-infused every 30 min at a dose of 10 ml/kg. Modified ultrafiltration was used following separation from bypass. All patients received similar standard postoperative care in our institution.

### Statistical analysis

The sample size calculations are based on a serum concentration of cTnI at post-reperfusion time points of 4 hours of 0.60 ng/mL for the postconditioning group and of 0.75 ng/mL for the control group according to results of preliminary studies. With an *α* level of 0.05 and a test power of 0.80, the resulting sample size was 40 patients for each group. A risk of loss of patients to follow up of 1-5% was assumed.

Statistical analysis was performed using the SPSS 13.0 statistical software package. Data are expressed as means ± standard deviation for continuous variables, and frequencies were measured for categorical variables. The unpaired *t*-test or *t’*-test according to homogeneity test for variance was used to compare measurement data and *Fisher’s* exact test was used to compare enumeration data. Repeated-measure analysis of variance was used to evaluate differences over time within groups for cTnI. All *p* values <0.05 were considered to be statistically significant.

## Results

### Study population

From January 2010 to December 2011, after obtained approval by the ethic committee and written informed consent, a total of 99 consecutive patients who met the inclusion criteria were randomly divided either into a postconditioning group (n=49) or into a control group (n=50). Among them, 10 patients were excluded (infective endocarditis in 5 patients, TOF with absent left pulmonary artery in 3 patients, and postoperative residual VSD in 2 patients). Finally, the outcome analysis was conducted on 44 patients in the postconditioning group and 45 patients in the control group.

### Clinical outcomes

The preoperative clinical data are shown in Table 1. No significant differences were noted with regard to age, gender, weight, McGoon ratio, LVEDVI, LVEF, cardiac output, hemoglobin, and saturation of arterial blood oxygen between two groups. The operative and postoperative data are shown in Table 2. Patients from the two groups were similar with regard to the types of procedure, duration of bypass as well as aortic clamping, reoperation for bleeding, and the first postoperative 24-hour urine volume, blood transfusion volume, drainage volume and serum creatinine level. The first postoperative 24-hour inotropic drug score and use of furosemide as well as its intravenous infusion dose in the postconditioning group was significantly less than those in the control group (5.7±2.4 versus 8.4±3.6, p<0.0001; 20.5% versus 42.2%, p=0.0395; 1.2±0.3 versus 1.7±0.4 mg/kg, p=0.0027). Patients received morphine-induced postconditioning were less likely to present short duration of mechanical ventilation and ICU stay as compared to control patients (20.6±6.8 versus 28.5±8.3 hours, p<0.0001 and 40.4±10.3 versus 57.8±15.2 hours, p<0.0001, respectively). Only one patient in the control group died of pulmonary infection on the 11th day after surgery. Pre- and post-operative LVEF and cardiac output are shown in Table 3. Morphine-induced postconditioned patients had higher LVEF as well as cardiac output on the first postoperative day (0.57±0.15 versus 0.51±0.13, p=0.0467 and 1.39 ± 0.25 versus 1.24 ± 0.21 L/min, p=0.0029, respectively) as compared to the control group.

**Table 1:** Demographic and clinical features

	**Postconditioning (n=44)**	**Control (n=45)**	**p**
Age (years)	4.2±1.8	4.4±1.5	0.5702
Gender (male/female)	26/18	25/20	0.8312
Weight (kg)	13.6±4.5	14.1±4.7	0.6096
McGoon ratio	1.33±0.20	1.38±0.25	0.3010
LVEDVI (ml/m^2^)	32.8±4.5	33.4±5.3	0.5667
LVEF	0.62±0.17	0.64±0.15	0.5575
Cardiac output (L/min)	0.94±0.21	0.97±0.24	0.5323
Hemoglobin (g/L)	183±34	175±28	0.2285
SaO_2_ (%)	74±16	78±13	0.1985

LVEDVI, left ventricular end-diastolic volume index; LVEF, left ventricular ejection fraction; SaO_2_, saturation of arterial blood oxygen.

**Table 2:** Operative and postoperative characteristics

	**Postconditioning (n=44)**	**Control (n=45)**	**p**
Operative type			
Transannular patches (%)	33 (75.0%)	30(66.7%)	0.4858
CPB time (min)	85±17	83±15	0.5575
Aortic cross-clamp time (min)	61±12	58±12	0.2415
Rhythm recovery			
Spontaneous ^a^	40 (90.9%)	36 (80.0%)	0.2297
Defibrillation ^b^	4 (9.1%)	9 (20.0%)	
Redo surgery for bleeding	1 (2.3%)	1 (2.2%)	1.0000
First postoperative 24 hours			
Blood transfusion volume (ml/kg/24 h)	14±3	15±3	0.1195
Drainage volume (ml/kg/24 h)	17±4	18±5	0.3010
Peak inotropic score	5.7±2.4	8.4±3.6	<0.0001
Urine volume (ml/kg/24 h)	66±14	61±13	0.0843
Creatinine (mg/dl)	0.8±0.3	0.8±0.2	1.0000
Use of furosemide	9 (20.5%)	19 (42.2%)	0.0395
Dose of furosemide (mg/kg/24 h)	1.2±0.3	1.7±0.4	0.0027
Duration of mechanical ventilation (h)	20.6±6.8	28.5±8.3	<0.0001
Duration of ICU stay (h)	40.4±10.3	57.8±15.2	<0.0001
Mortality	0	1 (2.2%)	1.0000

^a^ Heart beat restored after aortic cross-clamp removal without electric defribrillation; ^b^ Heart beat restored after aortic cross-clamp removal with one or more electric defribrillation.

CPB, cardiopulmonary bypass; LVEF, left ventricular ejection fraction; ICU, intensive care unit.

**Table 3:** Pre- and post-operative LVEF and cardiac output

	**Postconditioning (n=44)**	**Control (n=45)**	**p**
**LVEF**			
Baseline data	0.62±0.17	0.64±0.15	0.5575
Postoperative first day	0.57±0.15	0.51±0.13	0.0467
Before discharge	0.63±0.18	0.60±0.16	0.4080
**Cardiac output** (L/min)			
Baseline data	0.94±0.21	0.97±0.24	0.5323
Postoperative first day	1.39±0.25	1.24±0.21	0.0029
Before discharge	1.55±0.31	1.47±0.28	0.2046

### Level of plasma cTnI

**Figure 1:**
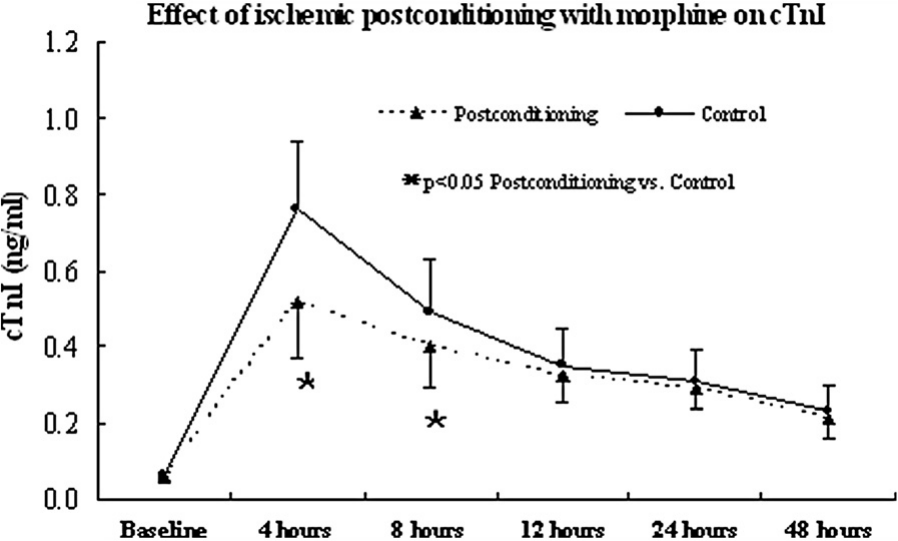
**Changes in levels of plasma cTnI over time.** (Postconditioning: postconditioning group; Control: control group; cTnI: cardiac troponin I).

As shown in Figure 1, serum concentration of cTnI increased sharply after surgery, reaching its peak within 4 hours, afterward only to be followed by a slow decline in both groups (time effect, F=61.45, p=0.000 for the postconditioning group and F=215.37, p=0.000 for the control group). Significant differences between the two groups were found at post-reperfusion time points of 4 hours and 8 hours (0.57 ± 0.15 versus 0.75 ± 0.20 ng/mL, p<0.0001 and 0.44±0.13 versus 0.50±0.12 ng/mL, p=0.0261, respectively). The AUC_0-48_ and AUC_0-8_ of cTnI release for the postconditioning group were significantly reduced as compared to the control group (14.68±3.52 versus 17.31±4.76, p=0.0040 and 3.06±0.93 versus 4.52±1.12, p<0.0001, respectively), although the AUC_8-48_ of the two groups were alike (11.62±2.59 versus 12.79±3.64, p=0.078).

## Discussion

The important finding of this trial was that morphine-induced postconditioning was related to less cTnI release postoperatively. Results of this trial showed that levels of plasma cTnI at all time-points after reperfusion increased sharply and remained high for at least 48 hours in both groups, which meant myocardial injury occurred and lasted for at least 48 hours after reperfusion. Plasma cTnI levels hit their peak values at the post-reperfusion time point of 4 hours in both groups, demonstrating myocardial injury occurred mostly during the first 4 hours after reperfusion. The morphine-induced postconditioned patients showing significant reduction of cTnI levels at the time points of 4 and 8 hours, especially the AUC calculation over the first 48 hours after aortic cross-clamp removal as compared to the control patients, might imply that the morphine-induced postconditioned patients received less myocardium injury as compared to the control patients. So, morphine-induced postconditioning used in this trial was able in part to relieve myocardial injury in patients with TOF undergoing cardioplegic arrest. Since most of cTnI is eliminated in urine, urine output might significantly affect its serum level [8,19]. Consequently, we concomitantly investigated the patients’ urine output during their first 24 hours in ICU, and found no significant difference in urine output between the two groups. Therefore, we can infer that the differences in serum cTnI levels between the two groups, particularly the cTnI AUC levels, could not be related to urine output but rather reflected release of different concentrations of cTnI by the two groups at different time points in this trial.

Two important mechanisms of cellular damage have been associated with cardioplegic arrest during open heart surgery, namely ischemic injury and reperfusion injury. In this trial, morphine-induced postconditioning reduced postoperative peak release of cTnI at the post-reperfusion time point of 4 hours by 31.6% compared to the control group and total cTnI release by 47.7% during the first postoperative 8 hours, suggesting that morphine-induced postconditioning may help to reduce myocardial injury induced by ischemia. However, cTnI release in the postconditioning group was similar to the control group at the post-reperfusion time points of 12, 24 and 48 hours, and the AUC_8-48_ of the two groups were alike, which might indicate that the degree of myocardial injury induced by the reperfusion was similar in the two groups and that morphine-induced postconditioning had failed to reduce myocardial injury induced by reperfusion [8,11]. This result was consistent with ischemic postconditioning reported previously [7,9,19].

Another important finding of this trial was that the morphine-induced postconditioned patients received some clinical benefits. Morphine-induced postconditioning reduced the peak inotropic score as well as the use of furosemide and increased LVEF as well as cardiac output during the first postoperative day, which might imply that morphine-induced postconditioning was related to better postoperative hemodynamics. In addition, the duration of mechanical ventilation in the postconditioning group was much shorter than that in the control group, which might infer that morphine-induced postconditioning was related to better postoperative cardiopulmonary function. Less inotropic drug use, higher LVEF as well as cardiac output, and shorter duration of mechanical ventilation coincided with reduction of plasma cTnI release, which might contribute to shorter length of ICU stay in morphine-induced postconditioned patients undergoing corrections of TOF. Although its protective effects could not be translated into more obvious clinical benefits such as lower mortality, morphine-induced postconditioning really provided enhanced cardioprotection and produced some clinical benefits mentioned above.

There are several limitations of this trial. Firstly, some important clinical decisions (for example, inotropes) were decided by clinician at work instead of specific protocols, weakening the inotropic score as an important endpoint of this trial. Secondly, enrolled patients were in a low-medium risk condition with short duration of aortic cross-clamping. It remains to be determined if high-risk patients such as those with prolonged aortic cross-clamping may benefit more from morphine-induced postconditioning. Thirdly, fentanyl also is a widely used opioid along with morphine during general anesthesia, and fentanil-induced postconditioning also protects ischemia/reperfusion. The plasma concentrations of fentanil were not measured in this study. Finally, this study was only a clinical observational trial in a single centre. Effective myocardial tissue concentration of morphine was not detected in this clinical trial, and the precise mechanisms of morphine-induced postconditioning in humans were not explored. Multi-centre clinical trials involving larger sample size are needed to determine the clinical effect of morphine-induced postconditioning in pediatric cardiac surgery.

## Conclusions

In summary, this trial showed morphine-induced postconditioning before the removal of the aortic clamp was related to less cTnI release, decreased inotropic drug use, increased LVEF as well as cardiac output, and shorter duration of mechanical ventilation as well as ICU stay. This clinical observational trial suggested morphine-induced postconditioning may provide enhanced cardioprotection against ischemia/reperfusion injury in children undergoing corrections of TOF.

## Abbreviations

TOF: Tetralogy of Fallot; cTnI: Cardiac troponin I; LVEDVI: Left ventricular end-diastolic volume index; LVEF: Left ventricular ejection fraction; CPB: Cardiopulmonary bypass; ACC: Aortic cross clamping; ICU: Intensive care unit; AUC: Area under curve.

## Competing interests

The authors declare that they have no competing interests.

## Authors’ contributions

Rufang Zhang and Qiang Ji conceived of the study, and participated in its design and coordination and helped to draft the manuscript. Li Shen and Yewei Xie participated in the design of the study and performed the statistical analysis. Lin Gen carried out the biochemical analysis and drafted the manuscript. Xiaobing Li carried out the biochemical analysis. All authors read and approved the final manuscript.
